# Identification and characterization of the intercellular adhesion molecule-2 gene as a novel p53 target

**DOI:** 10.18632/oncotarget.11366

**Published:** 2016-08-18

**Authors:** Yasushi Sasaki, Miyuki Tamura, Kousuke Takeda, Kazuhiro Ogi, Takafumi Nakagaki, Ryota Koyama, Masashi Idogawa, Hiroyoshi Hiratsuka, Takashi Tokino

**Affiliations:** ^1^ Department of Medical Genome Sciences, Research Institute for Frontier Medicine, Sapporo Medical University, Sapporo, Japan; ^2^ Department of Oral Surgery, Sapporo Medical University, Sapporo, Japan

**Keywords:** ICAM2, p53, p53 family, cell invasion, cell migration

## Abstract

The p53 tumor suppressor inhibits cell growth through the activation of both cell cycle arrest and apoptosis, which maintain genome stability and prevent cancer development. Here, we report that intercellular adhesion molecule-2 (ICAM2) is transcriptionally activated by p53. Specifically, ICAM2 is induced by the p53 family and DNA damage in a p53-dependent manner. We identified a p53 binding sequence located within the *ICAM2* gene that is responsive to wild-type p53, TAp73, and TAp63. In terms of function, we found that the ectopic expression of ICAM2 inhibited cancer cell migration and invasion. In addition, we demonstrated that silencing endogenous ICAM2 in cancer cells caused a marked increase in extracellular signal-regulated kinase (ERK) phosphorylation levels, suggesting that ICAM2 inhibits migration and invasion of cancer cells by suppressing ERK signaling. Moreover, ICAM2 is underexpressed in human cancer tissues containing mutant p53 as compared to those with wild-type p53. Notably, the decreased expression of *ICAM2* is associated with poor survival in patients with various cancers. Our findings demonstrate that ICAM2 induction by p53 has a key role in inhibiting migration and invasion.

## INTRODUCTION

The p53 transcription factor is the most important tumor suppressor and is known as the “guardian of the genome”. The p53 protein is activated by DNA damage or other cellular stresses and the activated p53 exerts its tumor suppression function mainly through the transactivation of a large number of downstream target genes, many of which are involved in apoptosis, cell-cycle arrest, and DNA repair [1, 2]. Interestingly, p53 was demonstrated to modulate the expression of genes that can modify cell motility [3–5]. Therefore, identification of novel p53 target genes is key to understanding the role of p53 in carcinogenesis.

The p53 family is composed of a group of transcription factors, p53, p73, and p63 [6–8]. In contrast to p53, the dominant-negative ΔN-isoforms of p73 and p63 are overexpressed in several cancer types [9–11]. In general, the TA-isoforms may be expected to have a role in tumor suppression, whereas several studies have described oncogenic functions for ΔNp63 in squamous cell carcinoma [12, 13]. In order to discover novel cancer-related genes, we performed a comprehensive analysis of the target genes of the p53 family. Among these, the present study focuses on *ICAM2* as a candidate for novel target genes for all three p53 family members (p53, TAp73, and TAp63).

Four major families of cell adhesion molecules have been identified: integrin, cadherin, selectin, and immunoglobulin superfamilies. ICAM2 is a member of the immunoglobulin superfamily that binds to β2 leukocyte integrin, which is a leukocyte function-associated antigen-1 (LFA1) [14]. When expressed in endothelial cells, platelets, and lymphocytes, ICAM2/LFA1 interaction plays a critical role in lymphocyte recirculation and trafficking, in the antigen-specific immune response, and in other cellular interactions important for immune response and surveillance [15–19]. However, the expression and function of ICAM2 in tumor cells has not been well investigated. Here, we analyzed ICAM2 regulation by all three members of the p53 family, and functionally linked ICAM2 to cancer cell migration and invasion. We found that ERK activation was enhanced by ICAM2 siRNA. A small-molecule MEK inhibitor reduced the effect of ICAM2 siRNA on cancer cell migration and invasion, suggesting that ICAM2 inhibits cancer cell migration and invasion at least in part through the suppression of the MEK-ERK signaling pathway.

## RESULTS

### Upregulation of ICAM2 mRNA and protein by p53 family

**Figure 1:**
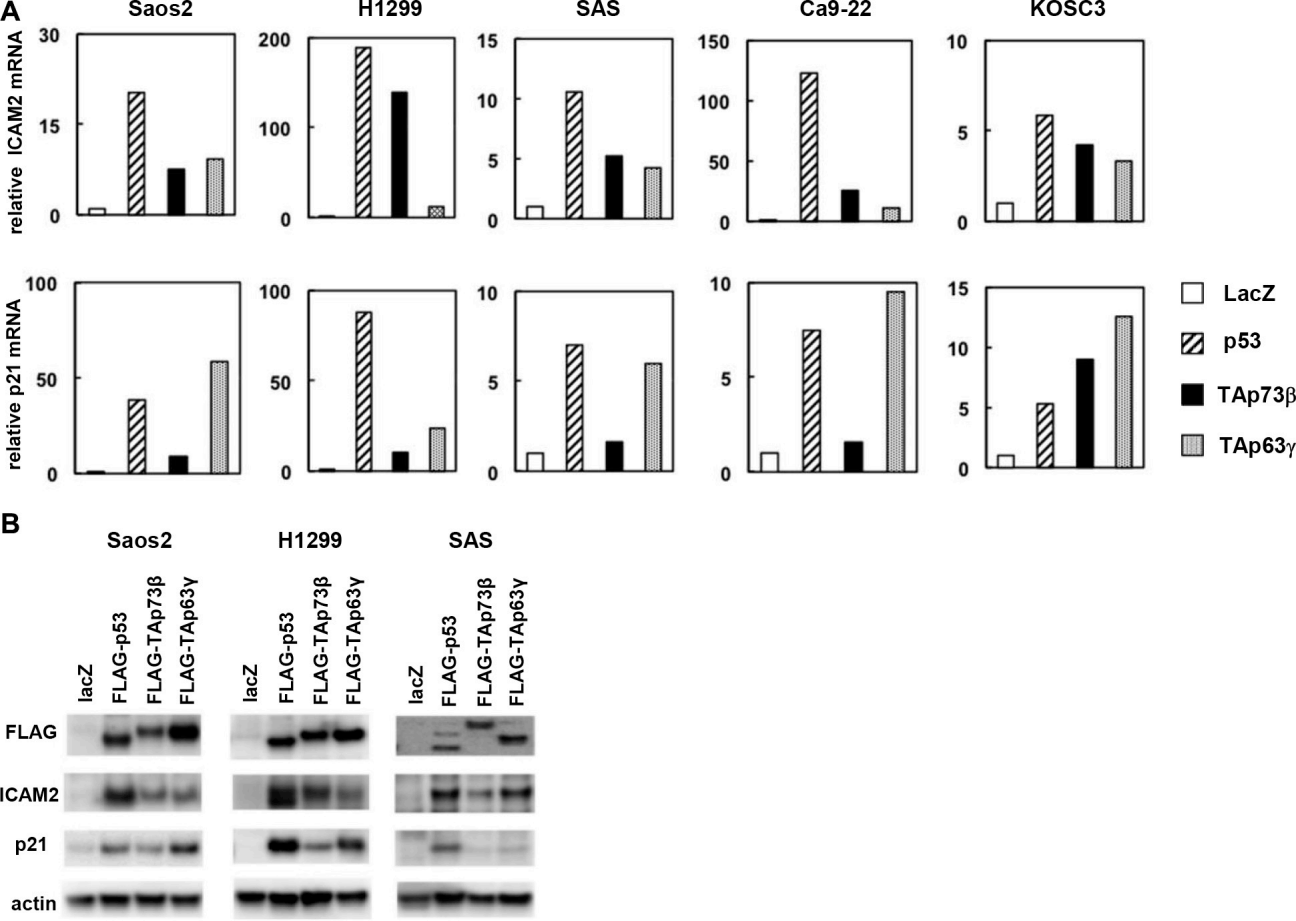
Upregulation of ICAM2 mRNA and protein by p53 family (**A**) Real-time RT-PCR analysis showed *ICAM2* mRNA upregulation following overexpression of the p53 family genes into human cancer cell lines. The cells were infected with adenoviruses expressing FLAG-tagged p53 family genes at an MOI of 25 (Saos-2 and H1299) or 100 (SAS, Ca9-22 and KOSC3), and the cells were harvested 24 h after infection. Relative gene expression levels were quantified using the ΔΔCt method and the results were normalized to the expression of the housekeeping gene *GAPDH*. The data are shown as the mean ± standard errors of three independent experiments and were normalized to their respective controls as 1. (**B**) Upregulation of ICAM2 protein by p53 family members. Cells were infected with the indicated adenoviruses as described above and immunoblot analysis of FLAG, ICAM2, p21, and β-actin was performed.

To identify the specific targets regulated by the p53 protein, we transfected human osteosarcoma cell line Saos-2 with adenoviral vectors expressing the p53 family members or LacZ as a control and compared mRNA expression by using a cDNA microarray. The microarray data were deposited into the NCBI Gene Expression Omnibus database (GEO; www.ncbi.nlm-nih.gov/geo) and are accessible through the GEO series accession number GSE13504. We validated strong induction of several known targets of the p53 family members, such as *p21*, *PIG3*, and *MDM2*, in this approach (please see GSE13504). Here, we focused on intercellular adhesion molecule-2 (ICAM2) as a candidate for novel p53 family targets. The results from the microarray were confirmed by real-time RT-PCR with five human cancer cell lines, Saos-2, H1299, SAS, Ca9-22, and KOSC3. *ICAM2* mRNA expression was clearly induced by p53, TAp73β, and TAp63γ in all of the tested cell lines (Figure 1A). In addition, the level of ICAM2 protein, as examined by immunoblot analysis, increased in p53-, p73-, and p63-transfected cells, consistent with the results of the real-time RT-PCR analysis (Figure 1B). As a positive control, we performed real-time RT-PCR and immunoblot analysis of the known p53 target, p21 (Figure 1A and 1B). We then examined whether activated endogenous p53 can induce ICAM2. We found that ICAM2 protein and mRNA were upregulated in HCT116-p53(+/+) cells treated with DNA-damaging agents, adriamycin (ADR) and 5-FU, and Nutlin-3, an inhibitor of MDM2. However, increased ICAM2 expression was not observed in HCT116-p53(−/−) cells following the same treatment. Because endogenous p73 and p63 proteins were not activated in both cells, the results indicated that ICAM2 is upregulated in an endogenous p53-dependent manner (Supplementary Figure S1).

### A responsive element for p53 family in the *ICAM2* gene

**Figure 2:**
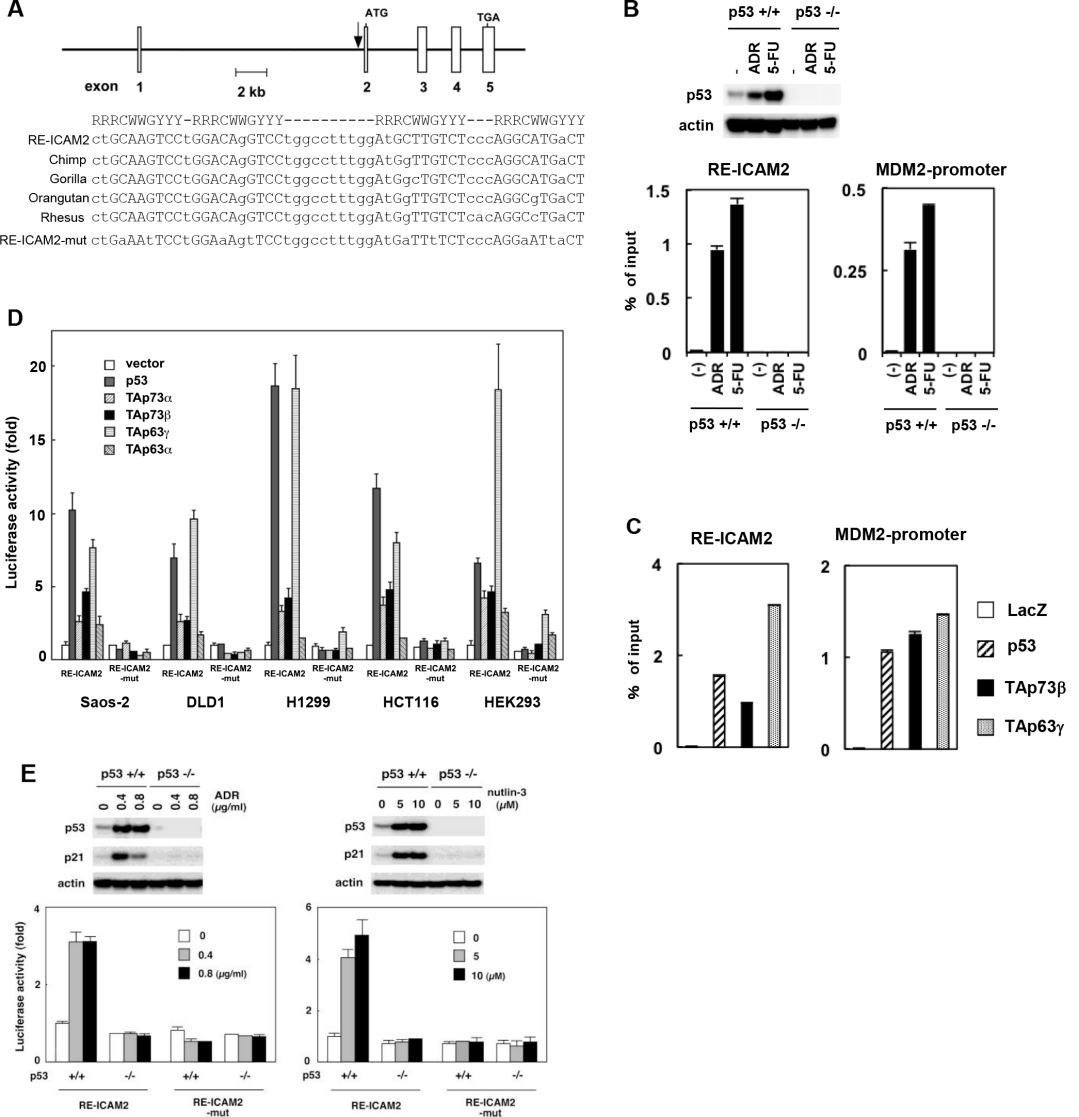
Regulation of *ICAM2* transcription by p53 family (**A**) The genomic position and sequence of the p53 response element within the *ICAM2* gene (RE-ICAM2). RE-ICAM2 is located within the first intron of the human *ICAM2* gene and consists of four copies of the 10-bp p53 consensus-binding motif. The alignment of the conserved binding sites in chimpanzee, gorilla, orangutan, and rhesus sequences from the *ICAM2* gene are shown. A mutated sequence corresponding to potentially critical nucleotides of RE-ICAM2 used in the luciferase assay is indicated in the bottom line (RE-ICAM2-mut). R represents purine; Y, pyrimidine; W, adenine or thymine. (**B**) Endogenous p53 binds to the RE-ICAM2 site *in vivo*. HCT116-p53(+/+) and HCT116-p53(−/−) cells were treated with 0.5 μg/mL ADR or 20 μg/mL 5-FU for 24 h and subjected to immunoblot analysis with an anti-p53 Ab (upper panels). ChIP assay for the presence of endogenous p53 protein at the RE-ICAM2 and *MDM2* promoter was performed. PCR amplifications were performed in triplicates for each precipitation with primers surrounding each site. The data were normalized to the signal from input DNA and the mean and standard deviation are indicated by the bars (lower panels). (**C**) p53 family proteins bind to the RE-ICAM2 site *in vivo*. Crosslinked chromatin was extracted from Saos-2 cells following infection with Ad-LacZ, Ad-p53, Ad-p73β, and Ad-p63γ, and the cell lysates were then immunoprecipitated with an anti-FLAG antibody. PCR amplifications were performed as described above. (**D**) The RE-ICAM2 sequence was responsive to p53 family members. Cells were transiently transfected with the pGL3-promoter vector containing RE-ICAM2 (pGL3-RE-ICAM2) or its mutant (pGL3-RE-ICAM2-mut) along with a transfection-control plasmid expressing Renilla luciferase, phRG-TK. Cells were co-transfected with a control vector or a vector that expresses p53 family members 24 h prior to performing the luciferase assay. Luciferase activity was measured using the dual-luciferase reporter assay system with the Renilla luciferase activity as an internal control. All of the experiments were performed in quadruplicates, and the mean and standard deviations are indicated by the bars. (**E**) HCT116-p53(+/+) and HCT116-p53(−/−) cells were co-transfected with the pGL3-RE-ICAM2 or pGL3-RE-ICAM2-mut together with phRG-TK. At 4 h after transfection, cells were treated with ADR or Nutlin-3 for 24 h and subjected to dual-luciferase assay. Experiments were done in quadruplicates with standard deviations indicated. Activity in the control HCT116-p53(+/+) cells was set to 1. The top panel shows western blot analysis of p53 and actin in cells examined from each luciferase assay.

To determine whether ICAM2 is a direct target of transcriptional activation by the p53 transcription factor, we searched for a consensus p53-binding sequence within the genomic locus encoding the human *ICAM2* gene and found four putative p53-binding sites (Supplementary Figure S2, left). We then performed chromatin immunoprecipitation (ChIP) assays to verify the direct binding of the p53 protein to candidate sequences using HCT116-p53(+/+) and HCT116-p53(−/−) cells. As expected, ADR or 5-FU treatment caused p53 activation in HCT116-p53(+/+) but not in HCT116-p53(−/−) cells (Figure 2B, upper panels). The ChIP assay revealed that endogenous p53 protein interacts with the chromatin region containing ICAM2-RE2/3 within the first intron of the human *ICAM2* gene in HCT116-p53(+/+) cells treated with DNA damaging agents (Supplementary Figure S2, right). This site consists of four copies of the 10-bp consensus p53-binding motif and is well-conserved among primate species at nearly identical positions within each orthologue (designated RE-ICAM2, Figure 2A). Furthermore, we confirmed the interaction between endogenous p53 and RE-ICAM2 in HCT116-p53(+/+) by real-time PCR (Figure 2B, lower panels). We also detected the interaction between p53 family member proteins and RE-ICAM2 (Figure 2C). As a positive control for the ChIP assay, we found that p53 family members can bind to the *MDM2* promoter (Figure 2B and 2C).

To determine whether the RE-ICAM2 sequences confer transcriptional activity in a manner that depends on the p53 family members, we performed a reporter assay using luciferase vectors including the RE-ICAM2 sequence (pGL3-RE-ICAM2). Cells were transiently co-transfected with pGL3-RE-ICAM2 together with a p53, TAp73α, TAp73β, TAp63γ, or TAp63α-expressing plasmid. As shown in Figure 2D, luciferase activity from pGL3-RE-ICAM2 increased in the presence of each of the tested p53 family members as compared with the control vector. In general, the largest isoforms, TAp63α and TAp73α, have weak activity to drive transcription. Luciferase activity from pGL3-RE-ICAM2 is higher in cells co-transfected with either p53 or TAp63γ than in those co-transfected with other p53 family members. In contrast, the mismatches in RE-ICAM2 (pGL3-RE-ICAM2-mut) significantly abolished transactivation by the p53 family. Next, we examined the RE-ICAM2 sequence to check whether it is responsive to transcriptional activation by endogenous p53. HCT116-p53(+/+) and HCT116-p53(−/−) cells were transiently transfected with pGL3-RE-ICAM2 or pGL3-RE-ICAM2-mut and incubated with ADR or Nutlin-3. The treatment resulted in the upregulation of p53 and its target p21 in HCT116-p53(+/+) cells. In addition, luciferase activities were significantly increased following ADR or Nutlin-3 treatment in HCT116-p53(+/+) cells (Figure 2E). In contrast, the same treatment did not substantially induce luciferase activity in HCT116-p53(−/−) cells. These data indicated that p53 directly transactivates the *ICAM2* gene through binding to RE-ICAM2.

### Effect of mutant-p53 and ΔN-isoforms of p53 family on ICAM2 expression

We then examined the ICAM2 levels following transfection with vectors containing tumor-derived p53 mutants. As shown in Supplementary Figure S3A, compared with the vector control, some p53 mutants (R175H, R249S, R273C and R282W) repressed ICAM2 protein expression in HCT116-p53(+/+) but not in HCT116-p53(−/−) cells. Downregulation of ICAM2 by mutant p53 was also observed in U2OS cells, in which endogenous p53 is wild-type. Additionally, ICAM2 induction by ectopic expression of wild-type p53 was partially reversed by mutant-p53 (Supplementary Figures S3B), indicating that certain p53 mutants indeed have a dominant negative effect against endogenous and exogenous p53 on ICAM2 induction. We then examined the effect of ΔN-isoforms of p53 family on ICAM2 expression. Endogenous ICAM2 protein was not induced in HCT116-p53(+/+) and HCT116-p53(−/−) cells following transfection with ΔN-isoforms of p53 family, such as ΔNp53, ΔNp63α and ΔNp63γ. Interestingly, ΔNp73β downregulated ICAM2 protein in HCT116-p53(+/+) but not HCT116-p53(−/−) cells (Supplementary Figure S4A).

### Silencing of ICAM2 promotes cancer cell migration and invasion

**Figure 3:**
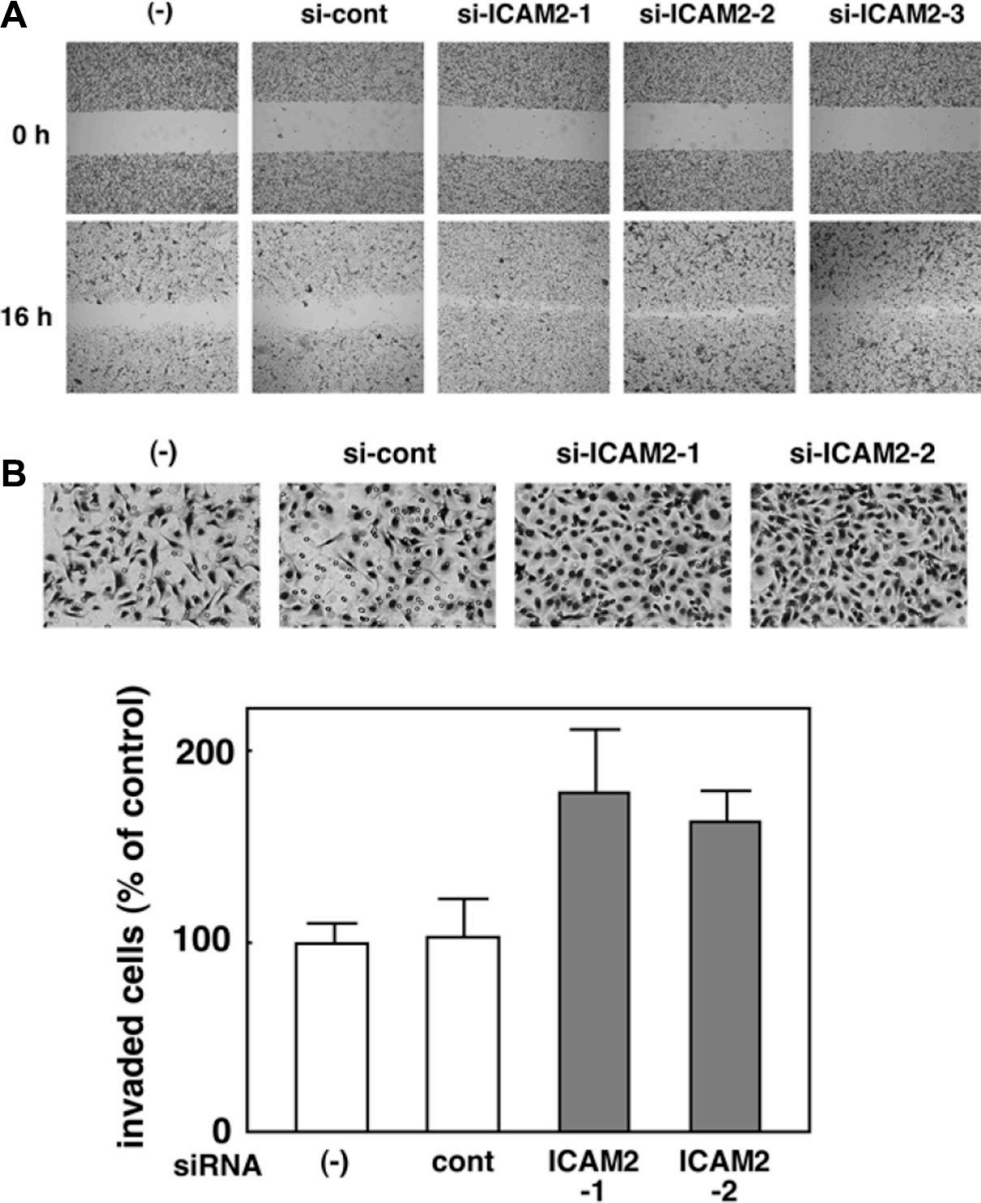
Silencing of ICAM2 by siRNA vector promotes cancer cell migration and invasion (**A**) Wound healing assay of ICAM2 siRNA plasmid-transfected cells. Phase contrast images were taken at 0 and 16 h after wounding. Representative images showed increased cell migration to the wounded area in ICAM2 siRNA-transfected HSC4 cells. (**B**) Cell invasion was measured in a Matrigel invasion assay following stable transduction. The experiments were repeated three times with similar results. Representative images showed increased cell invasion in ICAM2 siRNA-transfected HSC4 cells. Quantification of invasion as a percentage of the control is also shown.

We analyzed the endogenous protein expression of ICAM2 in a panel of cancer cell lines and found that ICAM2 expression appeared to vary across cell lines (Supplementary Figure S5). To further our understanding of the function of ICAM2, we stably knocked down ICAM2 with siRNA vectors targeting ICAM2 in HSC4 oral cancer cells, which express high levels of endogenous ICAM2. Three independent transfectants were established (si-ICAM2-1, 2, and 3) and ICAM2 protein expression was totally silenced in these 3 clones as opposed to parental cells and mock transfectants ((−) and si-cont, respectively, Figure 6A, upper panel). Cell viability was measured using the MTT assay. There was no major difference in proliferation between ICAM2-silenced cells and the control (Supplementary Figure S6), suggesting that ICAM2 has an insufficient effect on the cell proliferation. We next employed two different culture assays that include wound healing and Matrigel invasion assays. In the wound healing assay, we observed that downregulation of ICAM2 expression significantly increased cell migration (Figure 3A). To further investigate the effect of ICAM2 silencing on cell motility, Matrigel coated chambers were used to compare the invasiveness of HSC4 cells between the control and ICAM2 siRNA vector-transfected cells. ICAM2-silenced cells showed induced invasion over 1.5-fold in the HSC4 cell line (Figure 3B). Similar patterns were observed in the CHC-Y1 colorectal cancer cell line (Supplementary Figure S7). Additionally, HSC4 and CHC-Y1 cells exhibited fibroblastic, spindle-shaped morphology following silencing of ICAM2 (Supplementary Figure S8). Epithelial-mesenchymal transition (EMT) is a process by which epithelial cells gain migratory and invasive abilities. Because ICAM2 knockdown did not alter the expression of EMT markers including E-cadherin, N-cadherin and ZEB1. these results indicate that the downregulation of ICAM2 promotes cancer cell migration and invasion in an EMT-independent manner.

### ICAM2 expression inhibits cancer cell migration and invasion, and promotes cell adhesion

**Figure 4:**
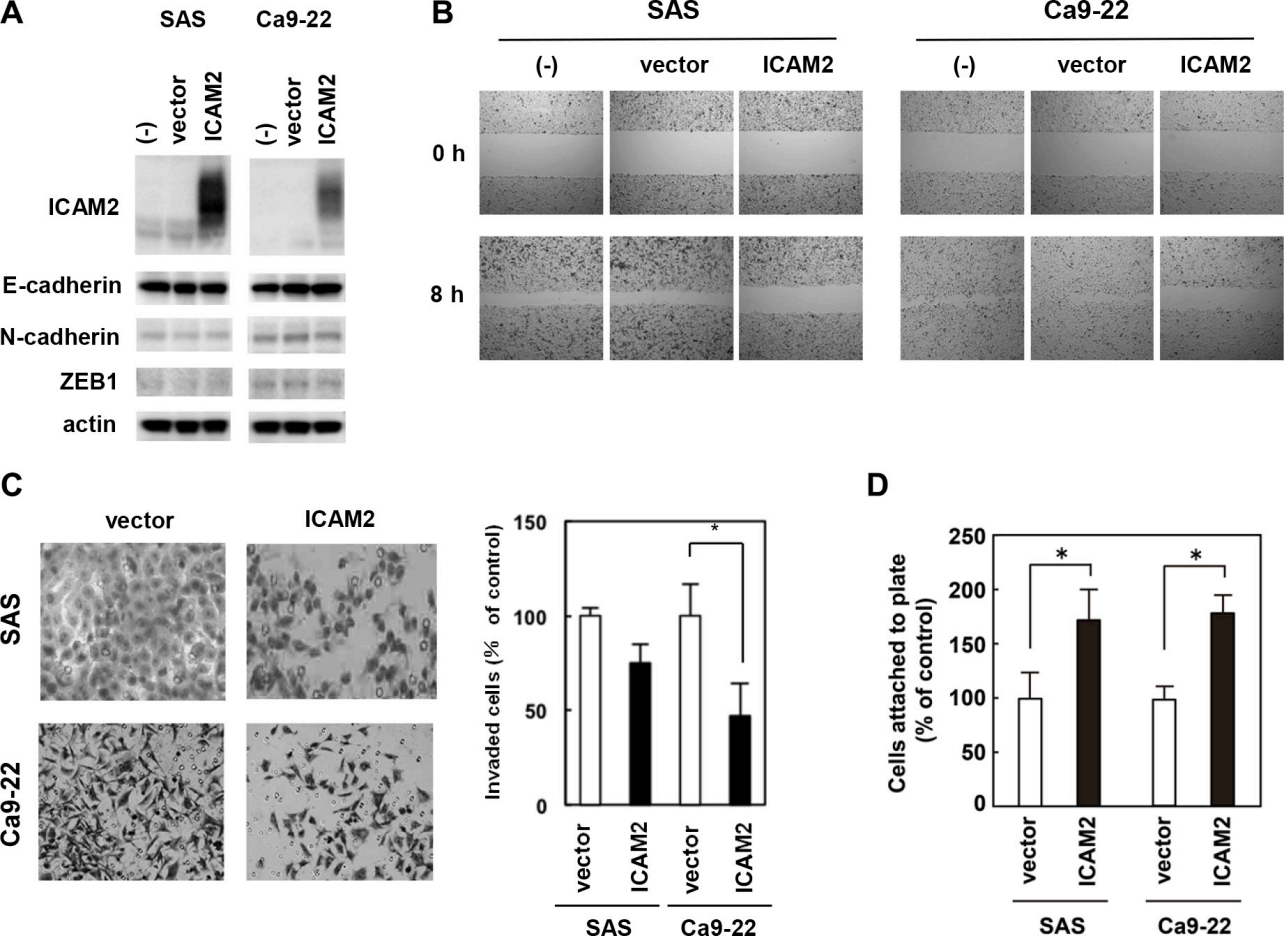
Overexpression of ICAM2 inhibits cancer cell invasion and migration, and promotes cell adhesion (**A**) Immunoblot analysis after stable transfection of ICAM2 expression vector or empty vector in SAS and Ca9-22 oral cancer cells. (**B**) Wound healing assay after stable transfection of ICAM2 expression vector or empty vector. Phase contrast images were taken at 0 and 8 h after wounding. Representative images showed increased cell migration to the wounded area in stably transfected SAS and Ca9-22 cells. (**C**) Cell invasion was measured in a Matrigel invasion assay following stable transfection of ICAM2 expression vector or empty vector. The experiments were repeated three times with similar results. Representative images showed decreased cell invasion in ICAM2-overwxpressing SAS and Ca9-22 cells (left). Quantification of invasion as a percentage of the control is shown (right). *p < 0.05; relative to empty vector (vector). (**D**) Cell adhesion was measured following transfection of ICAM2 expression vector or empty vector. Approximately 1 × 10^3^ cells were plated in triplicate on 96-well plates and incubated for 60 min at 37°C. The bound cells in each well were washed twice with PBS, stained with crystal violet, lysed with 2% SDS, and quantified by spectrophotometry at OD 595 nm. *p < 0.05; relative to empty vector.

**Figure 5:**
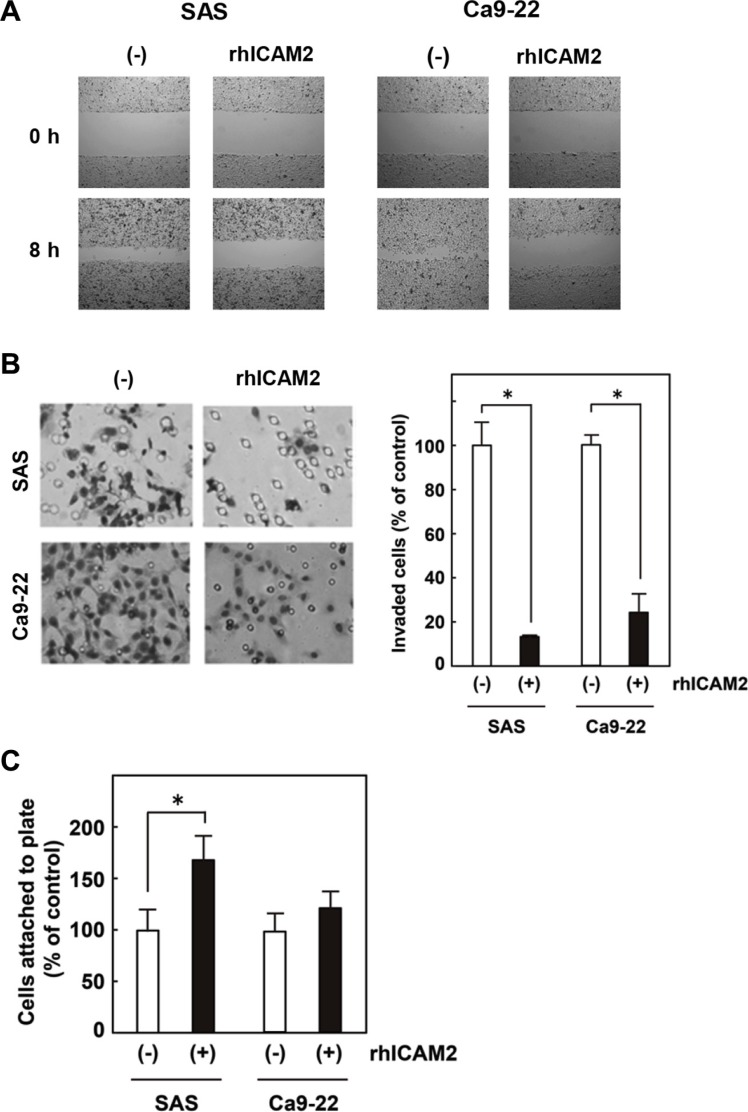
Recombinant human ICAM2 (rhICAM2) alters cancer cell migration, invasion, and adhesion (**A**) Wound healing assay after addition of rhICAM2 (62.5 ng/mL) to SAS and Ca9-22 cells. Phase contrast images were taken at 0 and 8 h after wounding. Representative images showing decreased cell migration to the wounded area after addition of rhICAM2. (**B**) Cell invasion was measured in a Matrigel invasion assay after addition of rhICAM2 to SAS and Ca9-22 cells. The experiments were repeated three times with similar results. Representative images showing decreased cell invasion after addition of rhICAM2 (left). Quantification of invasion as a percentage of the control is shown (right). *p < 0.05; relative to untreated cells. (**C**) Cell adhesion was measured after addition of rhICAM2. Approximately 1 × 10^3^ cells with or without rhICAM2 were plated in triplicates on 96-well plates. After 60 min incubation, cell adhesion was measured as described in the Figure 4D legend. *p < 0.05; relative to untreated cells.

We prepared a plasmid designed to express ICAM2 (pF5K-ICAM2) and performed wound healing and Matrigel invasion assays. SAS and Ca9-22 oral cancer cells were used; in both cells, endogenous ICAM2 expressions were at negligible levels (Figure 4A and Supplementary Figure S5). As shown in Supplementary Figure S9, no dramatic difference in proliferation between ICAM2-transfected cells and the control cells was observed, In contrast, forced ICAM2 expression significantly reduces the migration and invasion capabilities of these cancer cells (Figure 4A and 4B). Of importance, the migration-inhibiting effect of ICAM2 on Ca9-22 cells was completely neutralized by the addition of anti-ICAM2 neutralizing antibody (Ab) to the culture medium (Supplementary Figure S10A). Moreover, anti-ICAM2 Ab partially abrogated the inhibitory activity of ICAM2 on cancer cell invasion (Supplementary Figure S10B). Additionally, migration and invasion of SAS and Ca9-22 cells was significantly inhibited in response to recombinant ICAM2 protein (Figure 5A and 5B). These results suggest that p53-inducible ICAM2 regulates cancer cell migration and invasion. We also found that ICAM2 overexpression and the presence of recombinant ICAM2 protein enhanced cancer cell adhesion (Figures 4D and 5C).

### Regulation of the MEK-ERK pathway by ICAM2

**Figure 6:**
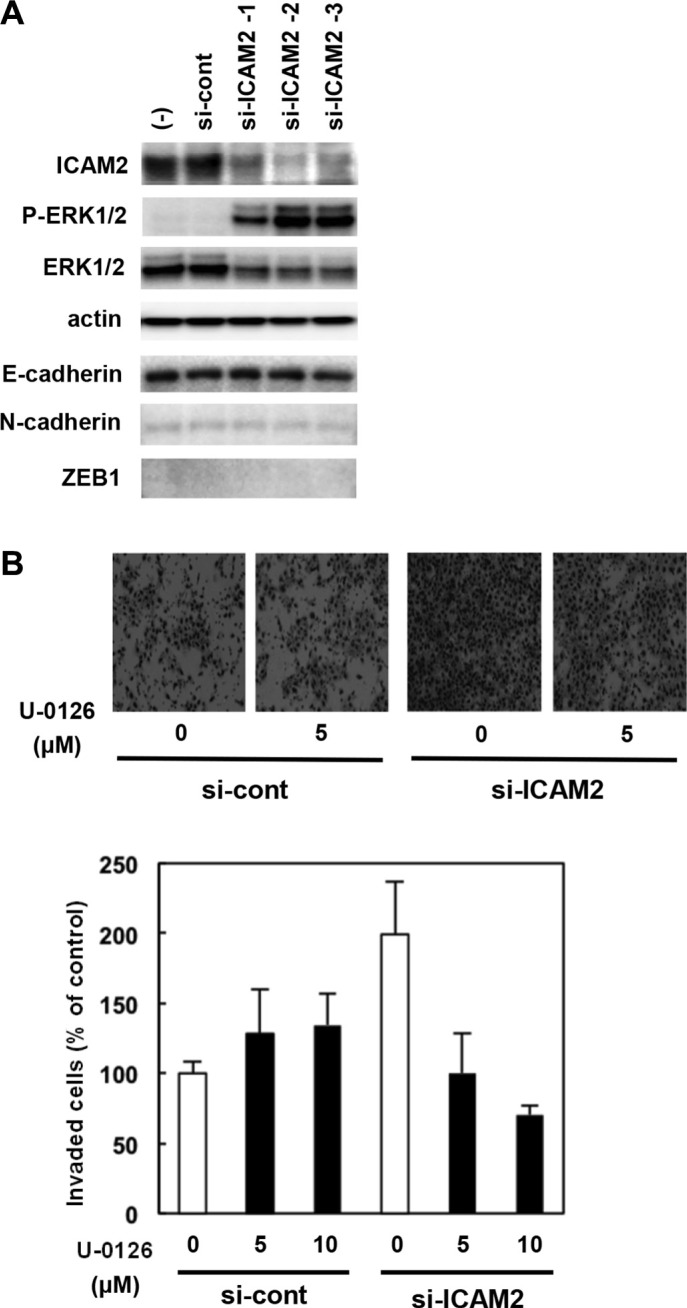
Regulation of the MEK-ERK pathway by ICAM2 (**A**) ICAM2 siRNA enhanced ERK1/2 phosphorylation in cancer cells. After stable transfection of ICAM2 siRNA plasmid (3 populations, si-ICAM2-1, 2, and 3) or empty plasmid (si-cont) in HSC4 oral cancer cells, cell lysates (20 μg of protein) were subjected to immunoblot with antibodies against p-ERK1/2, ERK1/2, and β-actin. (**B**) The invasion-enhancing effect of ICAM2 siRNA was inhibited by the addition of MEK inhibitor. Cells were suspended in medium containing 0, 5, or 10 μM U-0126 (0.5 mL/chamber) and then subjected to invasion assays. The experiments were repeated at least three times independently. Quantification of invasion as a percentage of the control is shown (lower panels).

Activation of extracellular signal-regulated kinases (ERKs) is known to lead cancer cell migration and invasion. Here, we show that the levels of the phosphorylated forms of ERK increased significantly by silencing ICAM2 in HSC4 (Figure 6A, 2nd panels), CHC-Y1, and DLD1 cells (Supplementary Figure S11A), when compared with parental cells and mock transfectants. Additionally, ICAM2 silencing enhanced FBS-induced phosphorylation of ERK in HCT15 colorectal cancer cells (Supplementary Figure S11B). We used the MEK inhibitor, U-0126, to address whether ERK activation is required for ICAM2 siRNA-mediated cancer cell invasion. In normal medium without U-0126, ICAM2-silenced HSC4 cells showed almost 2-fold increased invasion rate as compared to that in the control cells. This invasion-enhancing impact of ICAM2 siRNA was inhibited by the addition of U-0126 (Figure 6B). In contrast, U-0126 had less of an effect on control cell invasion. These results suggest that ICAM2 regulates cancer cell invasion, at least partially, through the ERK signal pathway.

### ICAM2 expression profiles in human tumors

We examined the relationship between *ICAM2* expression and p53 mutational status using the Oncomine Database (https://www.oncomine.org), in which RNA microarray and p53 mutation data have been reported. In six data sets, *ICAM2* expressions in tumors with mutant p53 were lower than those in tumors with wild-type p53 (Supplementary Figure S12), though this did not reach statistical significance. To examine whether *ICAM2* expression affects prognosis in cancers, we surveyed the PrognoScan database (http://www.prognoscan.org). Downregulation of *ICAM2* correlated with poor prognosis in certain cancers (breast, lung, bladder, and soft tissue cancers) (Supplementary Figure S13). This result indicates that low expression of *ICAM2* may serve as a poor prognostic factor for patients with cancers.

## DISCUSSION

The present study analyzed the mechanism of transcriptional regulation and tumor suppressor function of the p53 target candidate, ICAM2. When p53 family was introduced in multiple human cancer cell lines, the expression of ICAM2 gene and protein was induced (Figure 1). ICAM2 expression was also induced by the activation of endogenous p53 after DNA damage. We identified a consensus p53-binding sequence in intron 1of the human *ICAM2* gene and showed that the p53 family activates the transcription of ICAM2 through this sequence by using the ChIP and reporter assays (Figure 2). The results revealed that *ICAM2* is a new target gene for the p53 family.

*ICAM2* encodes a type I transmembrane glycoprotein that is a member of the ICAM family of adhesion proteins. ICAM2 is expressed in vascular endothelial cells and blood cells, and plays an important role in neutrophil migration into the tissue and cell-cell interactions during humoral immunity [14, 20]. Several reports state that ICAM2 functions as a tumor suppressor. ICAM2 facilitates an antitumor immune response by inducing the infiltration and accumulation of immature myeloid dendritic cells in pancreatic carcinogenesis [21]. ICAM2 downregulation has been shown in progression and metastasis of human neuroblastomas [22]. Furthermore, *in vitro*and mouse experiments indicate that ICAM2 downregulates the metastatic potential of neuroblastoma cells by interaction with the cytoskeleton proteins, such as α-actinin and actin [23, 24]. *ICAM1*, another member of ICAM family, has also been reported as the target gene that is controlled by p53 through direct transcription [25]. In the human fibroblast, expression of ICAM1 was increased during aging in a p53-dependent manner. ICAM1 expression correlated with p53 expression in the atherosclerotic lesions of human carotid arteries, indicating that ICAM1 is involved in p53-dependent senescence [26]. Taken together with our results, multiple members of ICAM family are transcriptionally activated by p53.

Our group have previously isolated a number of p53 targets, including genes involved in cancer cell migration and invasion. For example, we identified transcription factor FOXF1 as a direct p53 target, and FOXF1 inhibited cancer cell migration and invasiveness via controlling the expression of E-cadherin [27]. Similarly, a product of a p53 target gene, CLCA2, suppresses the motility of cancer cells through inhibition of the FAK transcription [28]. Loss of function of p53 results in dysregulation of its target gene expression, leading to abnormalities in the cell cycle and apoptosis, or in some cases, promotion of invasion and metastasis of cancer. p53 also controls EMT and stemness properties to suppress cancer cell invasion by through transactivation of its target genes [29]. In the present study, wound healing and Matrigel invasion assays were used to show that ICAM2 regulates the migration and invasion capacity of cancer cells. In addition, ICAM2 siRNA resulted in morphological alterations from cuboidal to fibroblastic shape (Supplementary Figure S8). Collectively, our findings indicate that ICAM2 suppresses cancer cell motility downstream of the p53 pathway.

The MEK/ERK pathway is activated by various growth factors and is an intracellular signaling pathway closely involved with cancer progression [30–33]. During the process of cell death, MEK/ERK pathway is intimately linked with the p53 signaling [34]. Importantly, p53 can transactivate target genes, which inhibit ERK activity [35, 36]. As shown in Figure 6, ERK activation was enhanced by silencing ICAM2 in several cancer cells, compared with parental cells and mock transfectants. MEK inhibitor, U-0126, reduced the effect of ICAM2 siRNA on cell invasion, suggesting that ICAM2 inhibits invasiveness of cancer cells through suppression of the MEK/ERK signaling pathway. ERK signaling is activated by extracellular stimuli such as growth factors and mitogens [37]. Here, cancer cell adhesion is also enhanced by ICAM2 overexpression or by the addition of recombinant ICAM2 protein to culture medium. The present results indicate that inhibition of ICAM2 may cause disruption of cell-cell contacts, leading to the ligand-dependent stimulation and aberrant ERK activation.

Some of the p53 mutants commonly found in human cancers can reduce expression of ICAM2 in cancer cells in a wild-type p53-dependent manner (Supplementary Figure S3). As clinical relevance of *ICAM2* expression, tumors with mutant p53 show lower expression of *ICAM2* mRNA than those with wild-type p53, although this did not reach statistical significance (Supplementary Figure S12). In contrast, we found no relationship between p53 mutation and *ICAM2* expression in cancer cell lines (Supplementary Figure S5), suggesting that ICAM2 is regulated by both p53-dependent and independent pathways. Furthermore, decrease in *ICAM2* expression was shown to be a poor prognostic factor in human breast, lung, and bladder cancers (Supplementary Figure S13). In a preclinical study, adenovirus-mediated transfer of the ICAM2 gene prolonged the survival of mice with peritoneal metastases of gastric cancer [38]. We found that neutralizing antibody against ICAM2 strongly promoted cancer cell migration and invasion (Supplementary Figure S10). On the other hand, similar to ICAM2 overexpression, addition of recombinant ICAM2 to culture media was sufficient to block cell migration and invasion. The activation of endogenous ICAM2 and exogenous administration of ICAM2 may be used as a treatment option for cancer. The p53-ICAM2-ERK pathway identified in the present study may become a new target of cancer treatment.

## MATERIALS AND METHODS

### Cell culture, recombinant adenoviruses, and plasmids

The human oral cancer cell lines SAS, Ca9-22, KOSC3, HSC4, osteosarcoma cell line Saos-2, lung cancer cell line H1299, and colorectal cancer cell line HCT116 were obtained from either the American Type Culture Collection or the Japanese Collection of Research Bioresources (Osaka, Japan). The HCT116 cell line (p53 wild type) and its derivative cell line HCT116-p53(−/−) lacking p53 were kindly provided by Dr Bert Vogelstein (Howard Hughes Medical Institute, Johns Hopkins University) [39]. The construction, purification, and infection procedures of replication-deficient recombinant adenoviruses encoding the human p53 family proteins fused to an amino-terminal FLAG epitope (Ad-p53, Ad-p73β, and Ad-p63γ) or the bacterial lacZ gene (Ad-lacZ) were performed as previously described. The relative efficiencies of the adenovirus infections into each cell line were determined by subjecting the cells infected with a control Ad-lacZ to X-gal staining and 90–100% of the cells were infected at an MOI of 25–100. To construct an ICAM2-expressing plasmid, the entire coding region of the human ICAM2 cDNA was inserted in-frame into the pF5K plasmid (Promega, Madison, WI, USA). We designed a small interfering RNA vector against human ICAM2. The iLenti-siRNA vector (Applied Biological Materials, Richmond, BC, Canada) containing the H1 promoter was used to express the siRNA. The targeted sequence of the iLenti-si-ICAM2 vector was 5′-CCACTTCACCTGCTCCGGGAAGCAGGAGT-3′. For the control, we used Lenti-siRNA vector without an insert. The anti-human ICAM2 neutralizing antibody (CD102) and recombinant human ICAM2 (rhICAM2) were purchased from R&D Systems (Minneapolis, MN, USA). Nutlin-3, ADR and 5-FU were purchased from Sigma (St Louis, MO, USA). U-0126 (Calbiochem, San Diego, CA, USA) was used at final concentrations of 5 and 10 μM.

### Real-time RT–PCR

RT–PCR was performed using TaqMan Gene Expression assays and a 7900HT real-time PCR system (Applied Biosystems, Carlsbad, CA, USA). Relative gene expression levels were quantified using the ΔΔCt method by normalizing transcript levels to the expression of the housekeeping gene glyceraldehyde-3-phosphate dehydrogenase (*GAPDH*). The data are shown as the mean ± standard errors of three independent experiments and were normalized to 1 based on their respective controls. The primer/probe sets used were as follows: ICAM2, Hs00609563_m1; CDKN1A (p21), Hs00355782_m1; and GAPDH, Hs99999905_m1.

### Immunoblot analysis

The primary antibodies that were used for immunoblotting were as follows: mouse anti-FLAG M2 monoclonal antibody (mAb) (Sigma), mouse anti-human ICAM2 (CD102, R&D Systems), mouse anti-human p21 mAb (F-5; Santa Cruz Biotechnology, Santa Cruz, CA, USA), mouse anti-β-actin mAb (C4; Chemicon, Billerica, MA, USA), mouse anti-human p53 mAb (DO-1; Santa Cruz Biotechnology), rabbit anti-human E-cadherin polyclonal Ab (H-108, Santa Cruz Biotechnology), rabbit anti-human ZEB1 polyclonal Ab (H-102, Santa Cruz Biotechnology), rabbit anti-human N-cadherin polyclonal Ab (H-63, Santa Cruz Biotechnology), and rabbit polyclonal Abs to phospho-ERK1/2 and ERK1/2 (Cell Signaling, Beverly, MA, USA). The proteins were transferred onto Immobilon-P membranes (Millipore, Billerica, MA, USA) by electroblotting and an immunoblot analysis was performed as previously described.

### Chromatin immunoprecipitation (ChIP) assay

The ChIP assays were performed by using the ChIP Assay Kit (Upstate Biotechnology, Lake Placid, NY, USA) and the antibodies against FLAG (M2). The cells (2 × 10^6^) were crosslinked with a 1% formaldehyde solution for 15 min at 37°C. The cells were then lysed in 200 μL of SDS lysis buffer and were sonicated to generate 300–800-bp DNA fragments. After centrifugation, the cleared supernatant was diluted 10-fold with the ChIP dilution buffer and was split into two equal portions; 1 portion was incubated with the appropriate antibody (5 μg) at 4°C for 16 h and the other portion was used as a negative control (no antibody). One-fiftieth of the volume of the total extract was used for PCR amplification as the input control. Immune complexes were precipitated, washed, and eluted, and DNA-protein crosslinks were reversed by heating at 65°C for 4 h. The DNA fragments were purified and dissolved in 40 μL of TE. The recovered DNA was submitted for PCR amplification using SYBR Green master mix and the 7900HT real-time PCR system. Relative binding values (% of input) were calculated using the comparative cycle threshold method (2^−ΔΔCt^). The PCR products generated based on the ChIP template were directly sequenced to verify the identity of the amplified DNA.

### Luciferase assay

A 54-bp fragment of the p53 response element in the *ICAM2* gene RE-ICAM2 (5′-CTGCAAGTCCTGGACAGGTCCTGGCCTTTGGA TGCTTGTCTCCCAGGCATGACT-3′) and its mutant form RE-ICAM2-mut (5′-CTGAAATTCCTGGAAAGT TCCTGGCCTTTGGATGATTTTCTCCCAGGAATTAC T-3′) were synthesized and inserted upstream of a minimal promoter in the pGL3-promoter vector (Promega). The resulting constructs were designated as pGL3-RE-ICAM2 and pGL3-RE-ICAM2-mut, respectively. Sub-confluent cells in 24-well plates were transfected with 2 ng of phRG-TK reporter (Renilla luciferase for internal control) and 100 ng of pGL3 reporter (Firefly luciferase as the experimental reporter), together with 100 ng of the appropriate expression vector by using the Lipofectamine 2000 reagent (Invitrogen, Carlsbad, CA, USA). Twenty-four hours after transfection, the reporter gene activities were measured using the Dual-Luciferase Reporter Assay (Promega) in accordance with the manufacturer's instructions. All of the experiments were performed in quadruplicates and were repeated three times.

### Cell invasion, migration and adhesion assays

Cell invasion was analyzed using Matrigel invasion chambers (Becton Dickinson, Franklin Lakes, NJ, USA) following the manufacturer's protocol. Briefly, cells were washed twice with PBS and were then resuspended in RPMI at the appropriate density according to the specific experiment. Equal volumes (0.5 mL/chamber) of the cell suspensions were seeded in the Matrigel invasion chambers (Becton Dickinson), which were placed in 24-well culture plates containing 0.75 mL/well of culture medium supplemented with 10% fetal bovine serum. Cells that had invaded to the underside of the inserts after 24-h incubation were stained and quantified by counting the cells from five microscopic fields. For cell migration assay, cells growing as a monolayer on collagen-coated coverslips were scratched manually with a plastic pipette tip. Wound healing was monitored at the indicated time points after scratching. For analysis of cell adhesion, 5 × 10^3^ cells were seeded on 96-well plates and incubated for 60 min at 37°C. The bound cells in each well were lysed, stained with 0.2% crystal violet, and quantified by spectrophotometry at OD 595 nm.

### Statistical analysis

Experimental data were evaluated using Student's *t*-test with probability values of < 0.05 considered significant.

## SUPPLEMENTARY MATERIALS FIGURES


