# Supplementary material for: Identification and characterization of the intercellular adhesion molecule-2 gene as a novel p53 target
Source: Oncotarget. 2016 Aug 18;7(38):61426–37. doi: 10.18632/oncotarget.11366 (PMC5308662; doi:10.18632/oncotarget.11366)
Supplement: Supplementary file 1 [file oncotarget-07-61426-s001.pdf]

Identification and characterization of the intercellular adhesion molecule-2 gene as a novel p53 target

Supplementary Materials

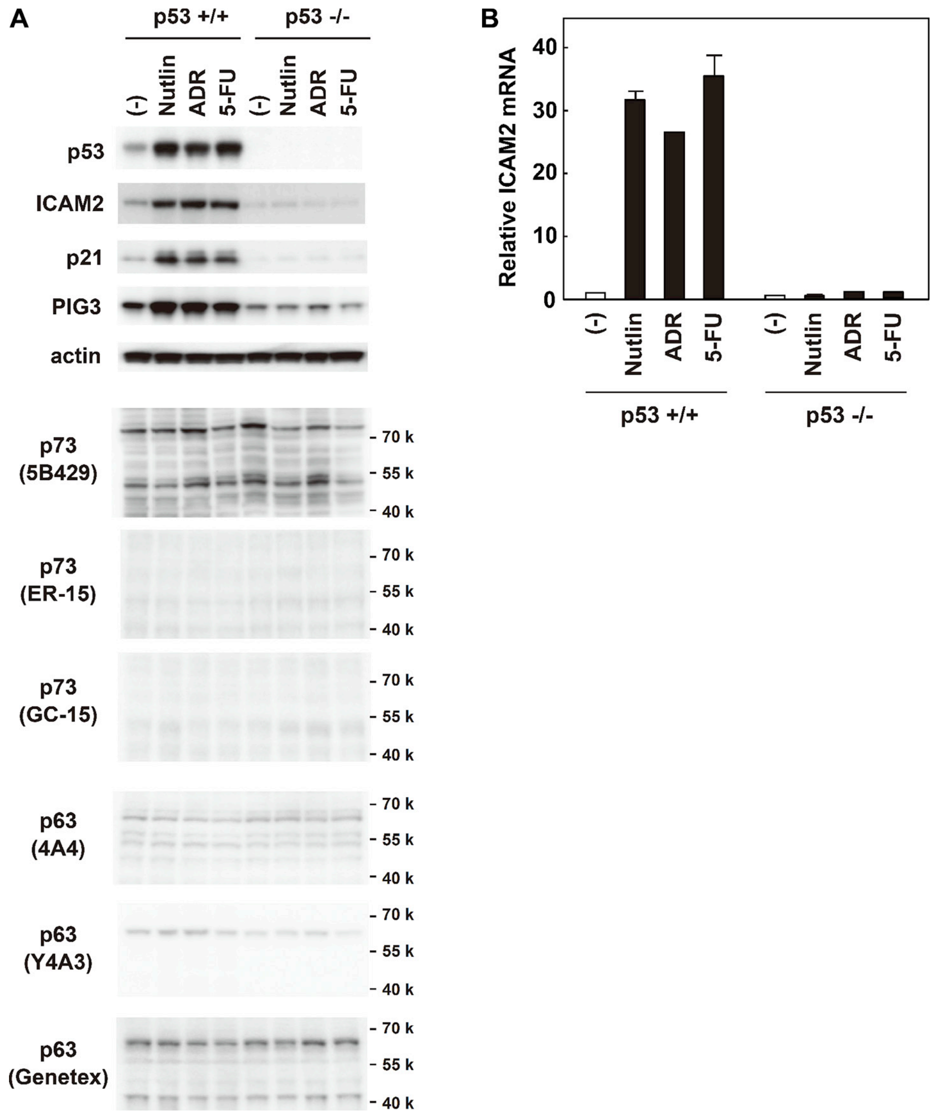

Supplementary Figure S1: Endogenous p53 upregulates ICAM2 protein and mRNA levels. (**A**) HCT116-p53(+/+) and HCT116-p53(−/−) cells were treated with 0.5 µg/mL ADR, 20 µg/mL 5-FU, or 10 µM Nutlin-3 for 24 h. Immunoblot analyses of ICAM2, p53, p21, PIG3, β-actin, p73, and p63 were conducted. The primary antibodies used for p73/p63 immunoblot are as follows: mouse anti-human p73 monoclonal antibody (mAb) (5B429, (Santa Cruz Biotechnology), ER-15 (Oncogene Research), and GC-15 (Oncogene Research)); mouse anti-human p63 mAb (4A4 (Santa Cruz Biotechnology) and Y4A3 (Thermo Scientific)); rabbit anti-human p63 polyclonal Ab (N2C1, Genetex Inc.). (**B**) Cells were treated as described above, and *ICAM2* mRNA levels were assayed by real-time RT-PCR. Relative gene expression levels were quantified using the ΔΔCt method and the results were normalized to the expression of the *GAPDH* gene. The data are shown as the mean ± standard errors of 3 independent experiments and were normalized to their respective controls as 1.

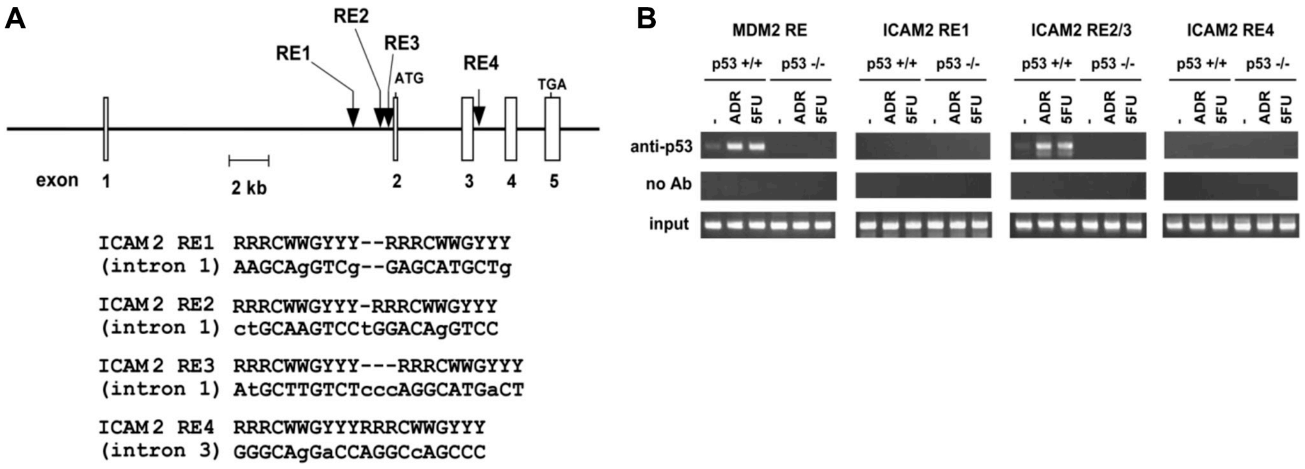

Supplementary Figure S2: Potential responsive sites in the *ICAM2* gene. (**A**) The position and nucleotide sequence of candidate response elements for p53 family in the *ICAM2* gene. The consensus sequences are indicated by upper-case letters and the spacer sequences between 10-bp motifs are indicated by -. Lower-case letters identify mismatches with the consensus sequence. R represents purine; Y, pyramidine; W, adenine or thymine. (**B**) HCT116-p53(+/+) and HCT116-p53(−/−) cells were treated with 0.5 μg/mL ADR or 20 μg/mL 5-FU for 24 h and subjected to ChIP assay in the presence of p53 protein at candidate binding sites and the *MDM2* promoter. Oligonucleotide primer sequences are as follows: ICAM2 RE1 sense 5′-GCGACACTTCAACCTTCTTGCC-3′, ICAM2 RE1 antisense 5′-CCCTGCCTCCCTGAGCTCCT-3′; ICAM2 RE2/3 sense 5′-TGCGTGTATGAGACGTGTGCA-3′, ICAM2 RE2/3 antisense 5′-AGCCTGGGCGACAAGAGTGAG-3′; ICAM2 RE4 sense 5′-GGTGCCCACCACCTTGCATAC-3′, ICAM2 RE4 antisense 5′-GGGTCCTTCAGACCAGCCTGC-3′; and *MDM2* promoter sense 5′- GTTCAGTGGGCAGGTTGACT-3′, *MDM2* promoter antisense 5′- GCTACAAGCAAGTCGGTGCT-3′. To ensure that PCR amplification was performed in the linear range, template DNA was amplified for a maximum of 40 cycles.

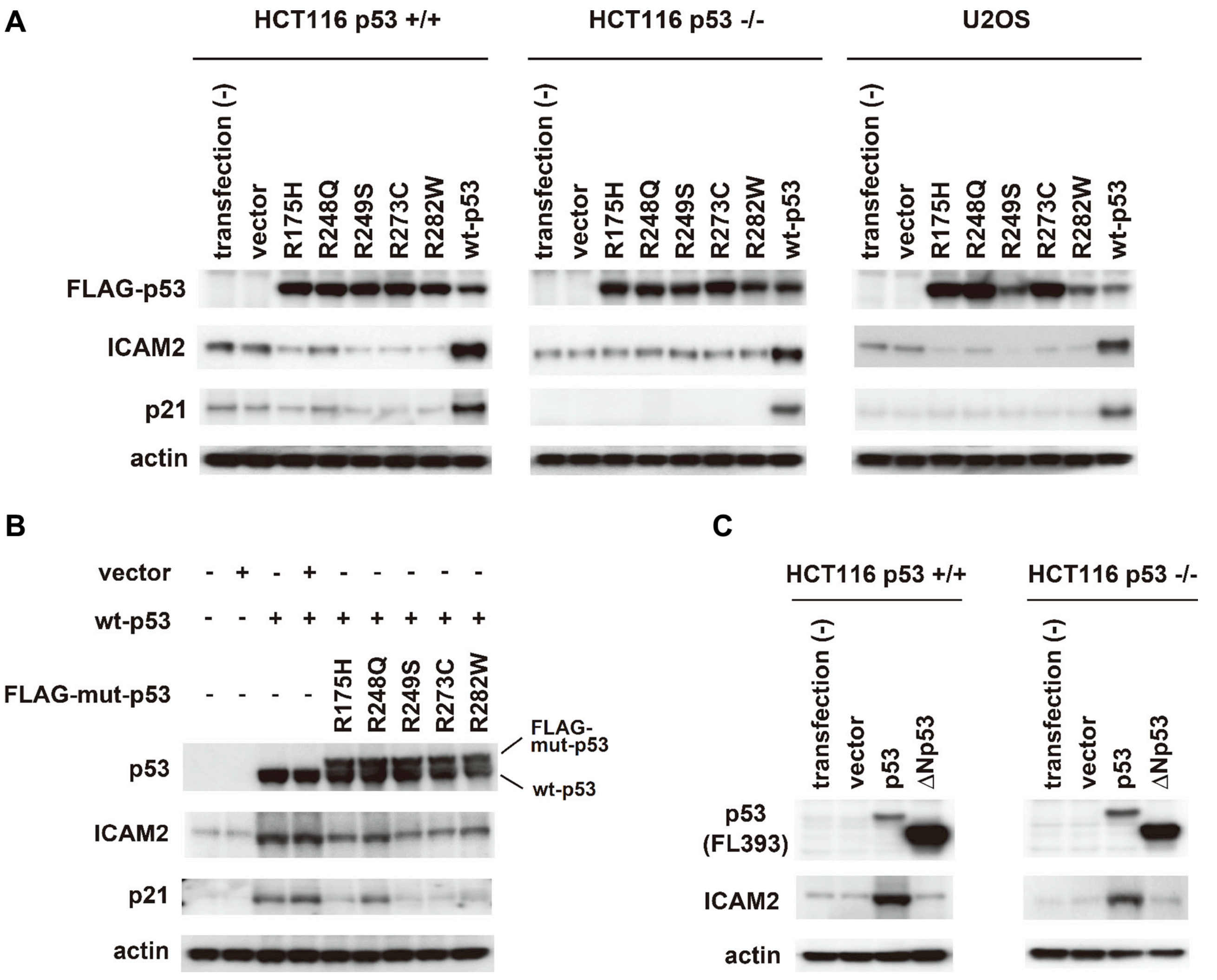

Supplementary Figure S3: The effect of mutant-p53 and ΔNp53 on ICAM2 expression. (**A**) Cells were transfected with various mutant p53–expressing plasmids or a wild-type p53-expressing plasmid. Cells were harvested 48 h after transfection, and immunoblot analysis was performed using an anti-ICAM2 Ab. p53 mutant constructs encoding p53 mutants commonly seen in human cancers (R175H R248Q, R249S, R273C, and R282W) were generated by subcloning into the pcDNA3.2 plasmid with an N-terminal FLAG epitope tag (Invitrogen). (**B**) HCT116-p53(−/−) cells were transfected with empty or FLAG-tagged mutant-p53-expressing vectors. After 8 h, cells were then transfected with wt-p53-expressing plasmid (no FLAG) for 24 h. ICAM2 induction by ectopic expression of wild-type p53 was partially reversed by all p53 mutants, indicating that certain p53 mutants indeed have a dominant negative effect against exogenous p53 on the regulation of ICAM2. (**C**) HCT116-p53(+/+) and HCT116-p53(−/−) cells were transfected with empty, wild-type p53-, or ΔNp53 (Δ40p53α)-expressing vectors and were harvested 24 h after transfection.ICAM2 and p53 protein levels were determined by immunoblot analysis . p53 immunoblot was performed using the rabbit anti-human p53 polyclonal Ab (FL393, Santa Cruz Biotechnology), which recognizes amino acids 1-393 of human p53. A ΔNp53 construct was generated by subcloning into the pF5K-CMV-neo plasmid (Promega).

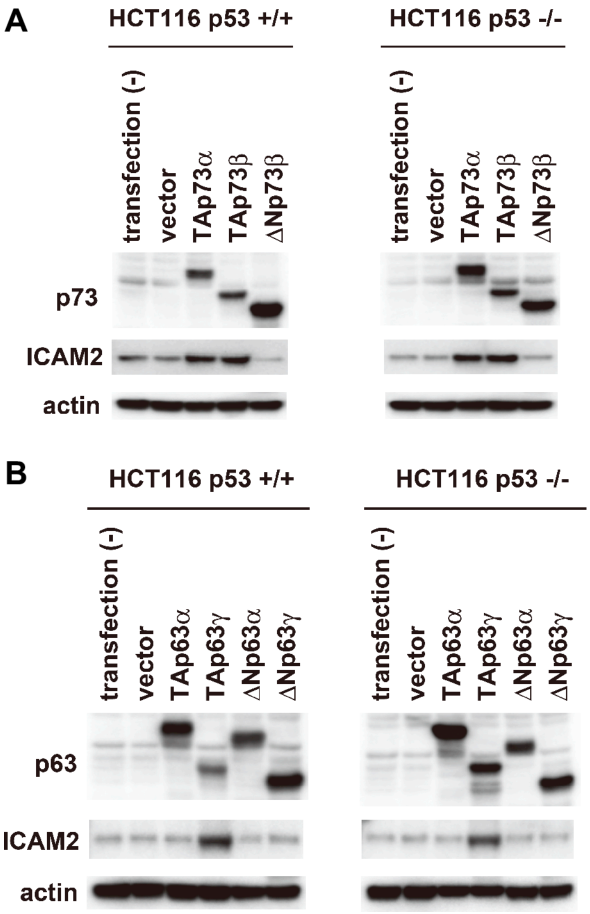

Supplementary Figure S4: The effect of ΔNp73 (A) and ΔNp63 (B) on ICAM2 expression. All ΔNp73/p63 constructs were generated by subcloning into the pF5K-CMV-neo plasmid. HCT116-p53(+/+) and HCT116-p53(−/−) cells were transfected with empty or p73/p63-expressing vectors for 24 h. ICAM2 protein levels were determined by immunoblot analysis.

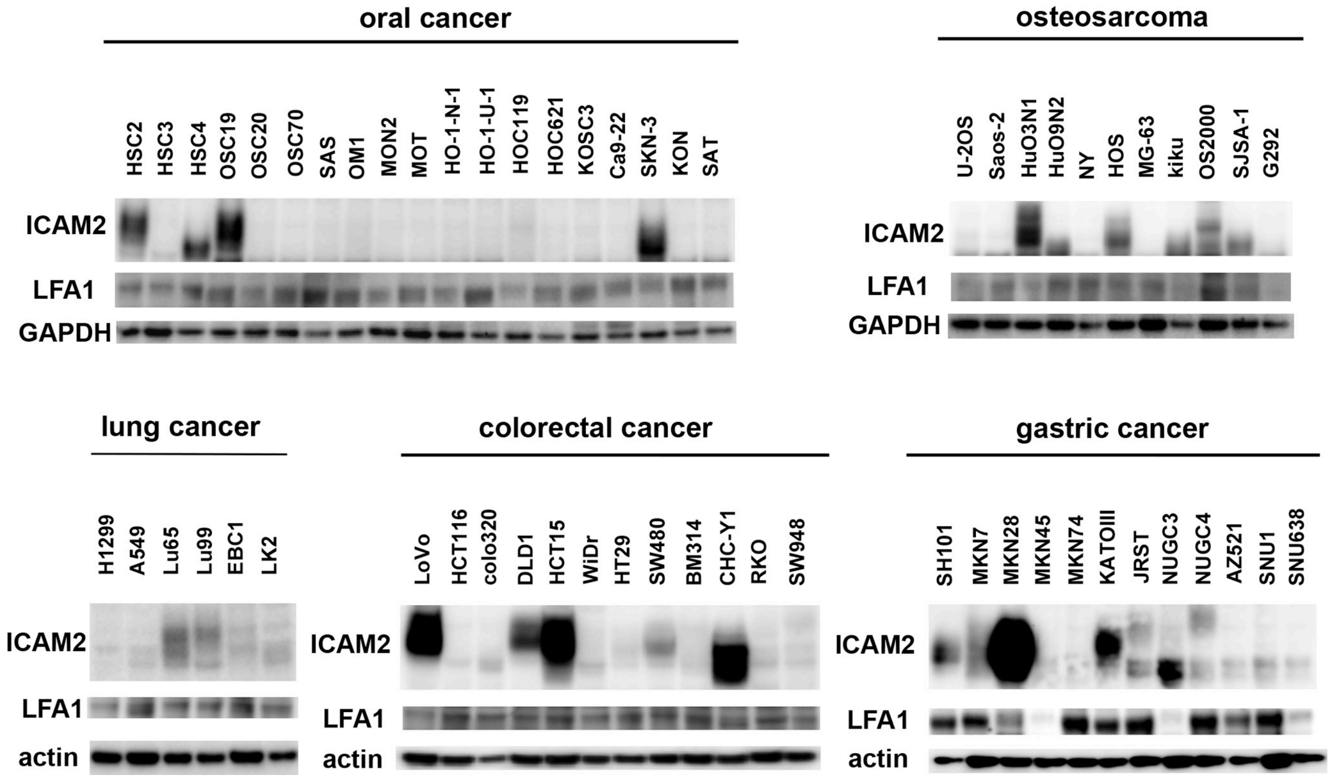

Supplementary Figure S5: Expression of endogenous ICAM2 and LFA1 in human cancer cell lines. Immunoblot analysis was performed using anti-ICAM2 and anti-LFA1 (CTB-104; Santa Cruz Biotechnology) antibodies. The endogenous p53 statuses in these cell lines are follows: Wild-type: U-2OS, HuO9N2, NY, MG-63, kiku, OS2000, SJSA-1, A549, Lu99, LoVo, HCT116, CHC-Y1, RKO, JRST, NUGC4, AZ521 and SNU1. Wt/mut: MKN45 Large deletion: Saos2, H1299 and KATOIII. Mutant-type: HSC2, HSC3, HSC4, OSC19, OSC20, OSC70, SAS, OM1, MON2, MOT, HO-1-N1, HO-1-U1, HOC119, HOC621, KOSC3, Ca9-22, SKN3, KON, SAT, HuO3N1, HOS, G-292, Lu65, EBC1, LK2, colo320, DLD1, HCT15, WiDr, HT29, SW480, BM314, SW948, SH101P4, MKN7, MKN28, MKN74, NUGC3 and SNU638. Data were from the IARC TP53 Database (http://p53.iarc.fr/CellLines.aspx), or we originally examined genetic alterations in exons 2 through 11 of the p53 gene (Oshima et al. Antitumor effect of adenovirus-mediated p53 family gene transfer on osteosarcoma cell lines. Cancer Biol Ther 2007; 6:1058–1066; Kusano et al. Genetic, epigenetic, and clinicopathologic features of gastric carcinomas with the CpG island methylator phenotype and an association with Epstein-Barr virus. Cancer 2006; 106:1467–1479; our unpublished data). Additionally, we performed a multigene sequencing screen interrogating 2855 mutational hotspots in 50 cancer-related genes using the AmpliSeq Cancer Hotspot Panel v2 and Ion Torrent semiconductor sequencer. This panel can explore hotspot regions of the following 50 cancer-associated genes: ABL1, AKT1, ALK, APC, ATM, BRAF, CDH1, CDKN2A, CSF1R, CTNNB1, EGFR, ERBB2, ERBB4, EZH2, FBXW7, FGFR1, FGFR2, FGFR3, FLT3, GNA11, GNAS, GNAQ, HNF1A, HRAS, JAK2, JAK3, IDH1, IDH2, KDR/ VEGFR2, KIT, KRAS, MET, MLH1, MPL, NOTCH1, NPM1, NRAS, PDGFRA, PIK3CA, PTEN, PTPN11, RB1, RET, SMAD4, SMARCB1, SMO, SRC, STK11, TP53, VHL. Library preparation and sequencing with an Ion Torrent PGM was performed as previously described (J Mol Diagn. 2013; 5:607–22). Approximately 800X average coverage was obtained with > 98% of target bases having at least 100 sequence reads. Alignment to the hg19 genome and variant calling was performed by Ion Reporter Software 4.0.

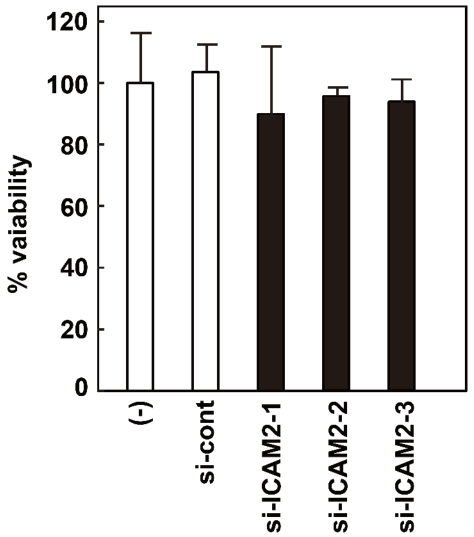

Supplementary Figure S6: ICAM2 silencing does not inhibit cancer cell growth. Expression of ICAM2 in HSC4 cells was silenced as described in the text. Cell viability was assessed by the uptake of tritium thymidine using a 3-(4,5-dimethylthiazol-2-yl)-2,5-diphenyltetrazolium bromide (MTT) assay. The assay was performed in triplicate with a Cell Counting Kit-8 (Dojindo, Tokyo, Japan) according to the manufacturer's instructions. Error bars equal one standard error.

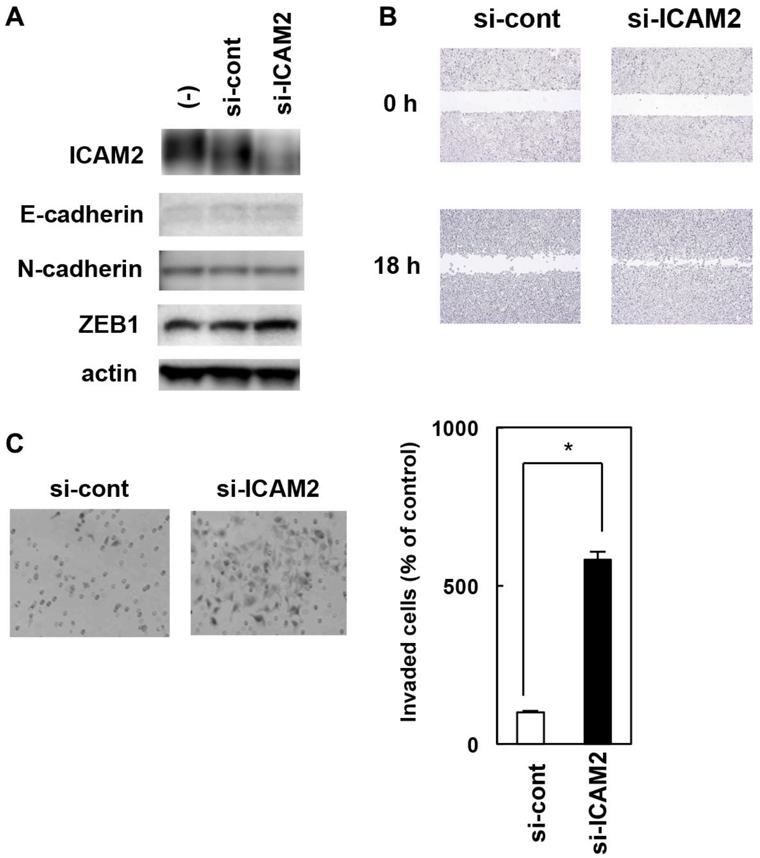

Supplementary Figure S7: Silencing of ICAM2 by siRNA vector promotes migration and invasion of CHC-Y1 cells. (**A**) Immunoblot analysis after stable transfection of ICAM2 siRNA plasmid (si-ICAM2) or empty plasmid (si-cont) in CHC-Y1 colon cancer cells. (**B**) Wound healing assay of ICAM2 siRNA plasmid-transfected cells. Phase contrast images were taken at 0 and 18 h after wounding. (**C**) Cell invasion was measured in a Matrigel invasion assay following stable transduction. The experiments were repeated three times with similar results. Representative images showed increased cell invasion in ICAM2 siRNA-transfected CHC-Y1 cells (left). Quantification of invasion as a percentage of the control is also shown (right). Asterisk, significant differences (**p* < 0.01; relative to empty vector (si-cont)).

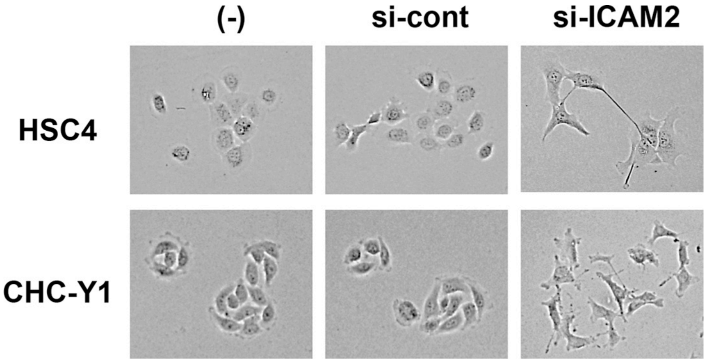

Supplementary Figure S8: Morphology analysis of HSC4 and CHC-Y1 cells following silencing of ICAM2 by siRNA vector. Photographs were taken under a phase-contrast microscope, showing that ICAM2-knockdown cells acquired a spindle cell type morphology with projections, and the number of cell-cell contacts was reduced.

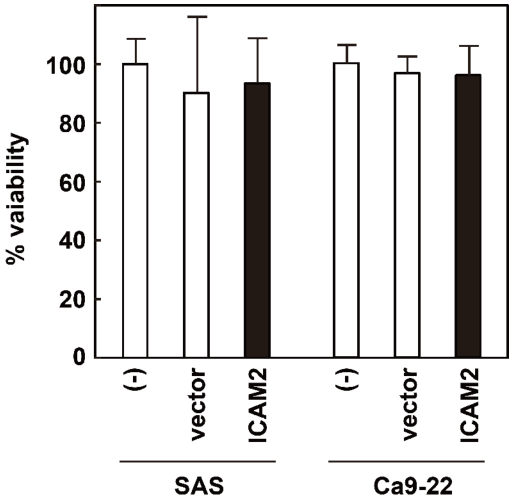

Supplementary Figure S9: Overexpression of ICAM2 does not inhibit cancer cell growth. SAS and Ca9-22 cells were stably transfected with ICAM2 expression vector or empty vector plasmid (vector). Cell viability was assessed by MTT assay. Error bars equal one standard error.

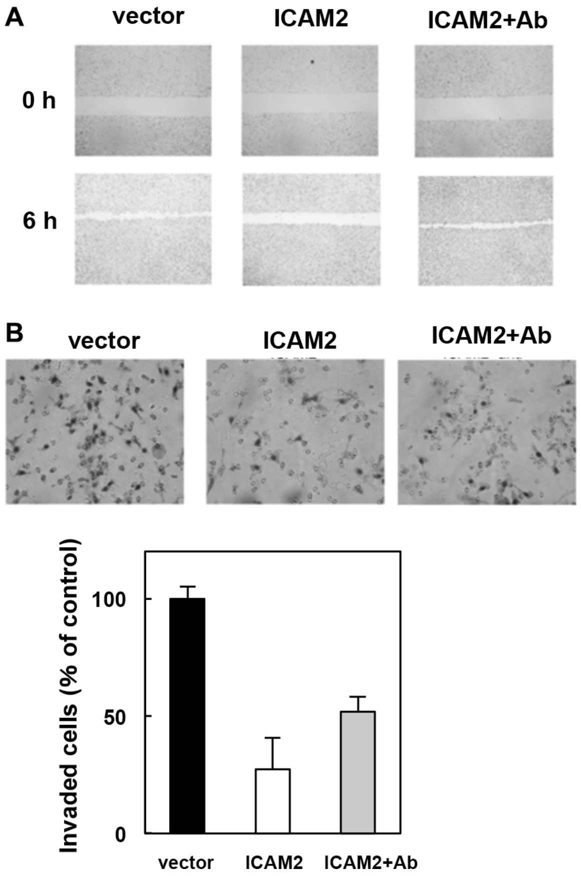

Supplementary Figure S10: ICAM2-mediated inhibition of cancer cell migration and invasion was blocked by anti-ICAM2 antibody. (**A**) The migration-inhibiting effect of ICAM2 was blocked by the addition of anti-ICAM2 Ab. Control and ICAM2-transfected Ca9-22 cell monolayers were scratched manually with a plastic pipette tip. Cells were washed with fresh medium to remove floating cells and cultured with or without anti-ICAM2 Ab (1.25 µg/mL). Wound healing was monitored 6 h after wounding. (**B**) The invasion-inhibiting effect of ICAM2 was blocked by the addition of anti-ICAM2 Ab. Control and ICAM2-transfected Ca9-22 cells were subjected to invasion assays. When necessary, an anti-ICAM2 Ab (1.25 µg/mL) was added to the medium. The experiments were performed in triplicates. Quantification of invasion as a percentage of the control is shown (lower panels).

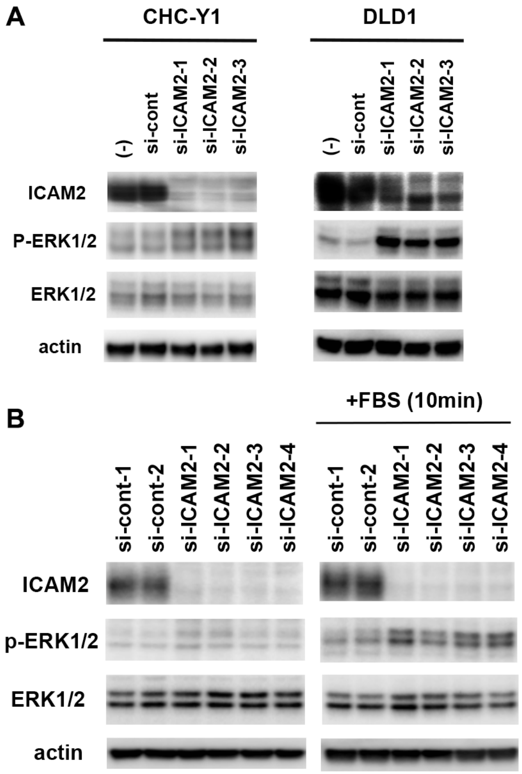

Supplementary Figure S11: ICAM2 siRNA enhanced ERK1/2 phosphorylation in cancer cells. (**A**) After stable transfection of ICAM2 siRNA plasmid (3 populations, si-ICAM2-1, 2, and 3) or empty plasmid (si-cont) in CHCY1 and DLD1 colorectal cancer cells, cell lysates (20 μg of protein) were subjected to immunoblot with antibodies against p- ERK1/2, ERK1/2, and β-actin. (**B**) HCT15 colorectal cells were stably transfected with ICAM2 siRNA plasmid (4 populations, si-ICAM2-1, 2, 3, and 4) or empty control plasmid (2 populations, si-cont-1 and 2). Cells were also serum-starved overnight, followed by FBS stimulation (10%, 10 min, right panel). Inhibition of ICAM2 expression by siRNA enhanced FBS-induced phosphorylation of ERK.

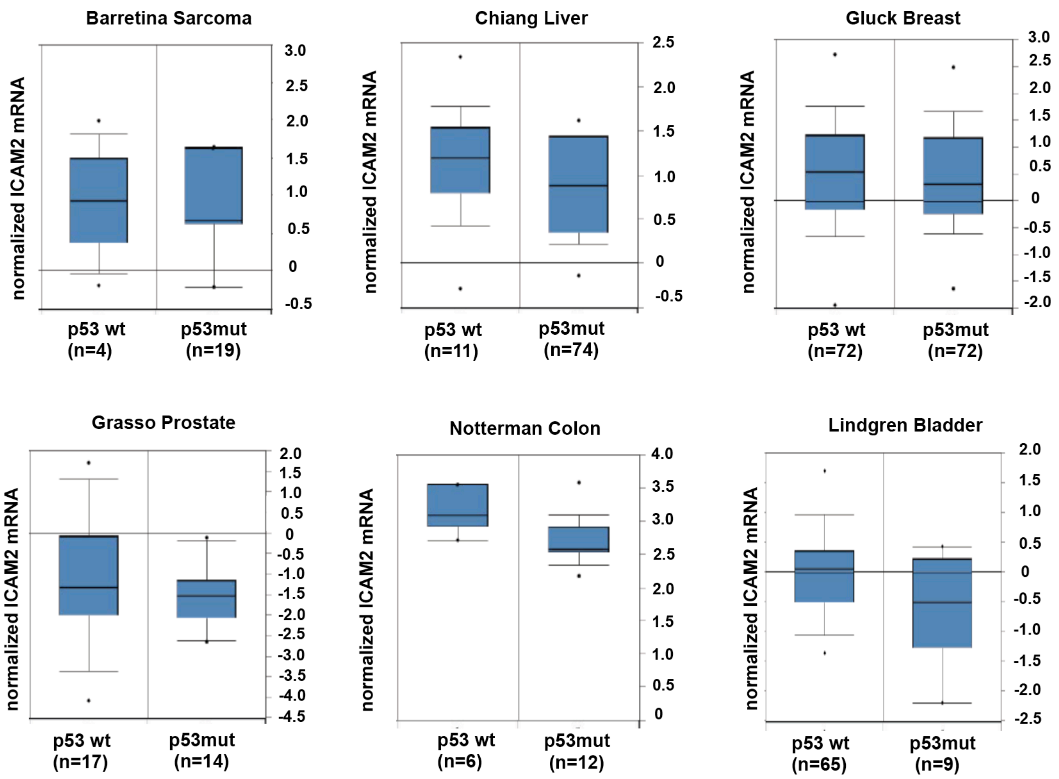

Supplementary Figure S12: *ICAM2* expression profiles in human tumors using published human oncology microarray data, Oncomine. Left bars, *ICAM2* expression in tumors with wild-type p53 ; right bars, *ICAM2* expression in tumors with mutant p53. Data are displayed as a boxplot (log2 median-centered) according to Oncomine output.

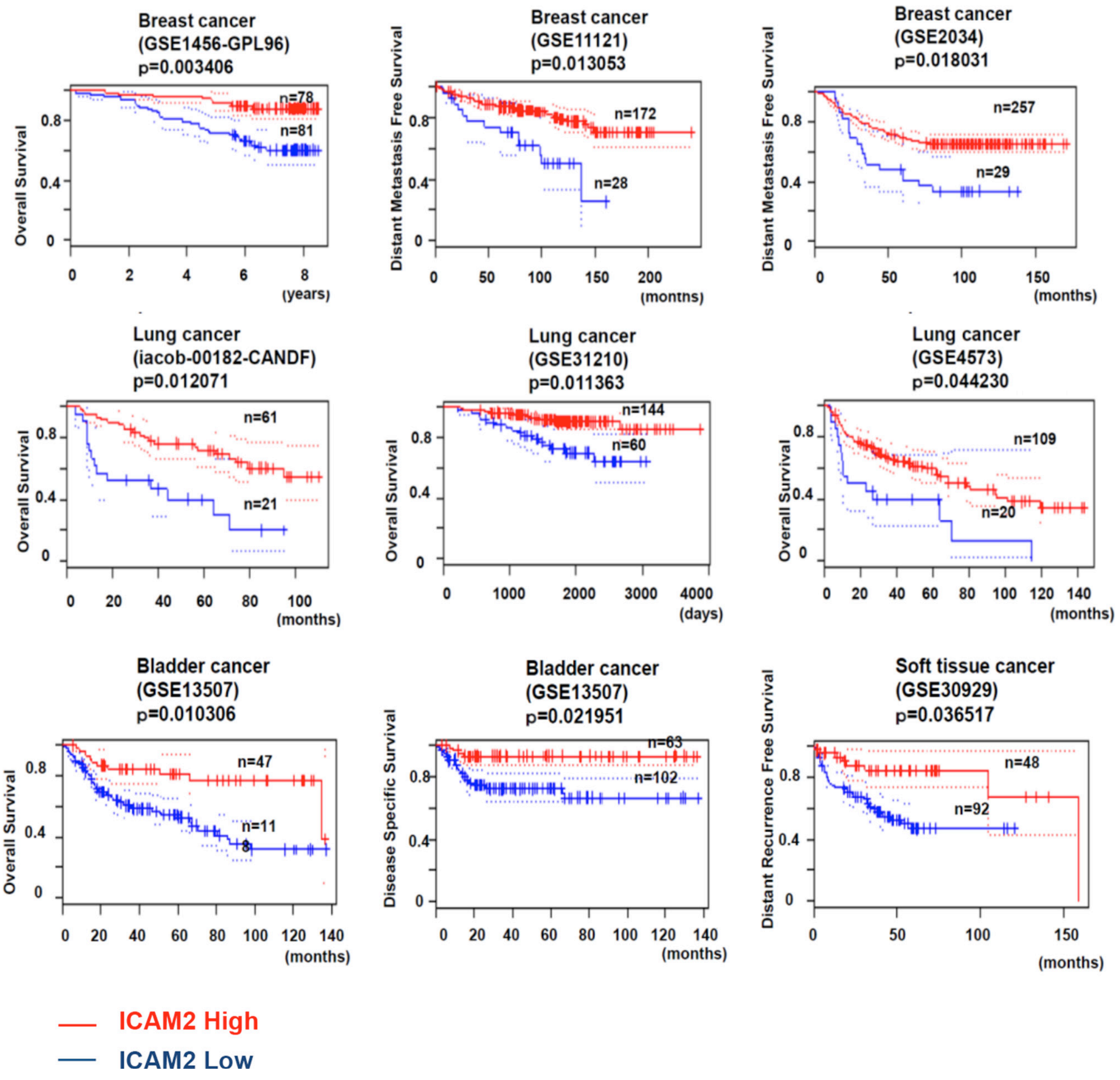

Supplementary Figure S13: The correlation between *ICAM2* expression and prognosis among cancer patients. Relationship between the expression of *ICAM2* and prognosis in cancer patients was examined using the PrognoScan database.
